# Urinary metabolomics reveals the biological characteristics of early pregnancy in pigs

**DOI:** 10.1186/s40813-022-00256-z

**Published:** 2022-03-21

**Authors:** Chen Zhou, Gengyuan Cai, Fanming Meng, Qun Hu, Guohao Liang, Ting Gu, Enqin Zheng, Zicong Li, Zhenfang Wu, Linjun Hong

**Affiliations:** 1 National Engineering Research Center for Breeding Swine Industry, College of Animal Science, South China Agricultural University, Guangzhou, 510642 Guangdong China; 2 Guangdong Provincial Key Laboratory of Agro-Animal Genomics and Molecular Breeding, College of Animal Science, South China Agricultural University, Guangzhou, China; 3 Institute of Animal Science, Guangdong Academy of Agricultural Sciences, Guangzhou, China

**Keywords:** Pig, Early pregnancy, Urine, Metabolome, Pregnanediol-3-glucuronide

## Abstract

**Background:**

Embryo implantation in sows is an important event during pregnancy. During this process, blastocysts undergo dramatic morphologic changes, and the endometrium becomes receptive. Studies have shown that developmental changes associated with the crosstalk between peri-implantation embryos and embryo-uterine are driven by various biomolecules secreted by the endometrium and embryos. In sows, changes in the uterus are also reflected in circulating body fluids and urine. Metabolomics reveals the metabolic state of cells, tissues, and organisms. In this study, we collected urine samples from large white sows during the peri-implantation period. The levels of urinary metabolites at different periods were analyzed using ultra-performance liquid chromatography/tandem mass spectrometry (UPLC–MS/MS) analysis techniques.

**Results:**

A total of 32 samples were collected from 8 sows during the estrus period and at each phase of early pregnancy (9, 12, and 15 days of gestation). A total of 530 metabolites were identified with high confidence in all samples. Compared with samples collected during the estrus phase, 269 differential metabolites were found in samples obtained during early pregnancy.

**Conclusions:**

The identified metabolites included lipids and lipid-like molecules, organic acids and their derivatives, organic oxygen compounds, organoheterocyclic compounds, benzenoids, among others. Metabolites, such as choline and pregnanediol-3-glucuronide, play important roles in pregnancy in sows and other animals. These results reveal the metabolic changes in urine of sows during early pregnancy phase. The differential urinary metabolites can be used for assessing peri-implantation status in sows. Understanding these metabolic changes may promote the management of pregnant sows through various interventions such as provision of proper nutrition.

**Supplementary Information:**

The online version contains supplementary material available at 10.1186/s40813-022-00256-z.

## Background

Early pregnancy in pigs is a complex process. The embryonic implantation stage (the first 15 days of pregnancy), is the phase of early pregnancy in sows. During this phase, the embryo and endometrium undergo strong morphological changes [1, 2]. At 8–10 days of gestation, the embryo freely moves throughout the uterine cavity, and gradually differentiates from a filamentous shape into a fetal shape [3]. At around 12 days of pregnancy, tubular embryos elongate rapidly within 1–2 h and become elongated filamentous embryos [4, 5]. This period is also called the peri-implantation period of pregnant sows [6]. By the 15th day of pregnancy, embryonic implantation begins [7, 8]. During this process, pig embryo moves between the uterine horns to provide enough space for attachment of the embryo [9]. During the embryonic attachment stage, steroid hormones are secreted [10], as well as many different preparations, such as growth factors, prostaglandins and cytokines [5], which assist in establishing a strong connection between the endometrium and the embryo. In the free blastocyst phase, embryo-maternal communication is mediated through complex signaling factors. The embryo-maternal communication is also fed back in body fluids, including urine as revealed in metabolomic studies [11]. Therefore, understanding the physiological characteristics of pigs during the early pregnancy period is particularly important to improve fertility.

Metabolomics refers to the study of metabolites and their chemical processes in biological samples. While a number of metabolites can be used as sources of metabolic information, mammalian urine provides some advantages as it contains a large number of metabolites [12] and can be non-invasively collected, thereby minimizing harmful impacts on animals [13]. In addition, as a humoral circulation metabolite, urine can better reflect all biochemical pathways in the body [14]. In recent years, most clinical assays have applied metabolomics in studies on pregnancy and pregnancy-related diseases like diabetes and ectopic pregnancy [15, 16]. Such studies have involved the use of maternal blood, amniotic fluid, and follicular fluid as metabolites [17, 18]. Elsewhere, mammalian urine has been used as a biomarker for studies on gestational diabetes mellitus and abortion [16]. It has been hypothesized that activities such as pregnancy induce metabolomic signatures that can be observed in mammalian urine [19]. However, there is little knowledge regarding urine metabolomic characteristics associated with the early pregnancy phase of mammals, especially pigs.

Pig embryos are too small to be prominent in the peri-implantation period. It is difficult to diagnose the pregnancy status from the appearance, or with the aid of B-ultrasound. Pig embryo implantation in early pregnancy is a multi-complex process that is affected by genetic, nutritional and environmental aspects. The effects of these processes may eventually be reflected in the final metabolites. Therefore, metabolomics analyses of sow urine in early pregnancy is a promising method for identifying the changes in metabolic biological characteristics in early pregnancy. If non-pregnant phenotypes are detected in early pregnancy phases, then, intervention measures can be taken to better improve reproductive efficiency and economic benefits. We used ultra-performance liquid chromatography–mass spectrometry to evaluate the differential changes in urine metabolites of sows in early pregnancy. The selected differential metabolites can be non-invasively used to assess the status of sows during the peri-implantation period. The differential metabolites revealed in this study can be used to model evidence for maternal-embryo communication as well as regulation and control strategies of maternal nutritional requirements for embryonic implantation during early pregnancy in pigs.

## Methods

### Study animals and sample collection

Eight clinically healthy Large White sows (parity 2) from the Wens Foodstuffs Group Co., Ltd (Yunfu, China) pig farm were selected for estrus synchronization treatment after which artificial insemination was performed. Midstream urine samples were collected from sows by the natural urination method on the 0th (DP0, non-pregnant, calculated on the day before insemination), 9th (DP9), 12th (DP12) and 15th (DP15) day of pregnancy. The collected urine samples were quickly placed in liquid nitrogen for metabolite investigation [20]. Immediate sample freezing was necessary to quench any rapid degradation activities, such as oxidation of labile metabolites as well as various enzymatic reactions [21, 22]. The pregnancy status of the sows were confirmed by B-ultrasound at the 4th and 6th weeks.

### Metabolite Extraction and quality control

Briefly, 200 μL urine samples were added into a 1.5 mL centrifuge tube using a pipette. An 800 μL acetonitrile:methanol (1:1, v/v) extraction solution was added to the content in the centrifuge tube, vortexed for 30 s at 5℃ and 40 kHz ultrasound for 30 min. Samples were maintained at − 20 °C for 30 min to precipitate the proteins. The supernatants were removed, centrifuged at 13,000×*g* for 15 min at 4 °C and dried using nitrogen. Then, they were reconstituted with 120 μL acetonitrile:water (1:1, v/v) reconstitution solution, ultrasonically extracted at 5 °C and 40 kHz for 5 min and centrifuged at 13,000×*g* at 4 °C for 5 min after which the supernatant was used for LC–MS/MS analysis [23].

To evaluate the stability of the analysis system during the on-boarding process, 20 μL of the supernatant was added to each sample and mixed as the quality control sample (Quality Control, QC). During instrumental analysis, a QC sample was injected for every 8 analytical samples. In data analysis, repeatability of the QC sample was used to investigate the stability of the instrument, and to find variables with large variations in the analysis system to ensure data reliability [24].

### UHPLC–MS/MS analysis

Chromatographic separation of metabolites was performed on a Thermo UHPLC system equipped with an ACQUITY BEH C18 column (100 mm × 2.1 mm I.D., Waters, Milford, USA). The mobile phase included 0.1% formic acid in water (solvent A) and 0.1% formic acid in acetonitrile: isopropanol (1:1, v/v) (solvent B). To balance the system, the solvent gradient varied according to the following conditions: from 0 to 3 min, from 95% (A): 5% (B) to 80% (A): 20% (B); from 0 to 3 min. At 3 to 9 min, 80% (A): 20% (B) to 5% (A): 95% (B); 9 to 13 min, and 5% (A): 95% (B) to 5% (A): 95% (B). From 13 to 13.1 min, 5% (A): 95% (B) to 95% (A): 5% (B), from 13.1 to 16 min, and 95% (A): 5% (B) to 95% (A): 5% (B). Sample injection volume was 2 μL at a flow rate of 0.40 mL/min. Column temperature was kept at 40 ℃. During analysis, these samples were stored at 4 ℃.

Mass spectra data were collected using the Thermo UHPLC-Q Exactive Mass Spectrometer equipped with electrospray ionization (ESI) sources operating in positive and negative ion modes, respectively. Optimum conditions were: Aus gas heater temperature 400℃, sheath gas flow 40 psi; Aus gas flow 30 psi; ion spray voltage floating (ISVF), negative mode-2800 V and positive mode 3500 V, normalized collision energy, MS/MS rolling 20–40–60 V. Data acquisition was performed via the Data Dependent Acquisition (DDA) mode. Detection was performed at the mass range of 70–1050 m/z.

### Metabolomics data processing

After UPLC-TOF/MS analysis, raw data was imported into Progenesis QI 2.3 (Waters, USA) for peak detection and comparisons. The preprocessing results generated a data matrix consisting of retention time (RT), mass-to-charge ratio (m/z) values and peak intensity. Online search was done using biochemical databases (Human Metabolome Database (HMDB) (http://www.hmdb.ca/) and Metlin database (https://metlin.scripps.edu/)), with cumulative mass, MS/MS fragment spectrum and isotope ratio differences. Mass spectrometry was used to identify these metabolic characteristics. For metabolites with MS/MS confirmation, only those metabolites with MS/MS fragment scores higher than 30 were considered credibly identified [25]. In the Progenesis QI workflow, we considered confidence scores higher than 50 sufficient for assignment of constituents in the iboga extract [26].

Multivariate statistical analyses were performed using ropls (Version1.6.2) R package from Bioconductor on Majorbio Cloud Platform (https://cloud.majorbio.com). Principle component analysis (PCA) using an unsupervised method was performed to obtain an overview of the metabolic data. Partial least squares-discriminant analysis (PLS-DA) and orthogonal partial least square discriminant analysis (OPLS-DA) was used for statistical analysis to determine the overall metabolic changes between comparable groups. Before OPLS-DA, all metabolite variables were scaled. Effectiveness of the model was evaluated from the model parameters, R2 and Q2, which provided information for interpretability and predictability of the model and avoided over-fitting risk. Variable Importance in Projection (VIP) was calculated in the OPLS-DA model. *p* values were determined using the paired Student’s t-test on single dimensional statistical analysis. Statistically significant groups with VIP values more than 1 and *p* values less than 0.05 were selected. The first 30 different metabolites were selected in the metabolic concentration of each pregnancy period, relative to the estrus period. Various metabolites between the two periods were summarized and mapped to their biochemical pathways through metabolic enrichment and pathway analysis based on database search (KEGG, http://www.genomic.jp/kegg/). Scipy.stats (Python package), using Fisher's exact test, was used to identify statistically significant enrichment pathways. In addition, the identified metabolite expression data (from DP0 to DP15 stage) were normalized to 0, log_2_^(DP9/DP0)^, log_2_^(DP12/DP0)^ and log_2_^(DP15/DP0)^, and the differential metabolites clustered by STEM [27].

## Results

### Untargeted metabolic profiling of urine during estrus and early pregnancy

**Fig. 1:**
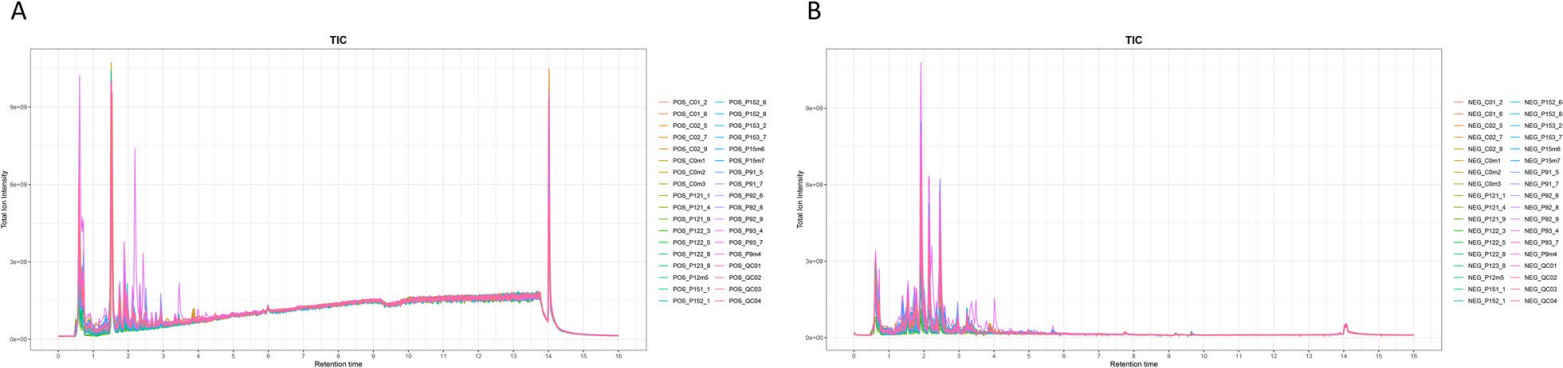
Typical total ion chromatograms (TICs), **A** positive ions and **B** negative ions, obtained from the UHPLC-Q Exactive ESI of Quality Control samples

To investigate the metabolic changes in urine during pregnancy in sows, non-targeted metabolomics analysis was performed. Chromatographic separation spectra showed good overlapping of ion chromatograms for each phase samples and quality control samples, in positive ion mode (Fig. 1A) and negative ion mode (Fig. 1B), after analysis by UPLC/Q-TOF MS. After data pre-processing, 5327 features were extracted from the positive ions. After normalization, 4660 features with relative standard deviation (RSD) of < 30% accounted for 87.48% of all QC samples (Additional file 2: Table S1). A total of 8747 features were extracted from negative ions. After normalization, 7560 features with RSD < 30% accounted for 86.43% of all QC samples (Additional file 3: Table S2). The correlation coefficient among the quality control samples was > 0.95. These results imply that there is no significant batch effect on the preprocessed data. Analysis of over 12,220 mass spectra recorded in the total ionization mode revealed 530 compound identifications with high confidence using Progenesis QI (score > 50, Additional file 4: Table S3).

**Fig. 2:**
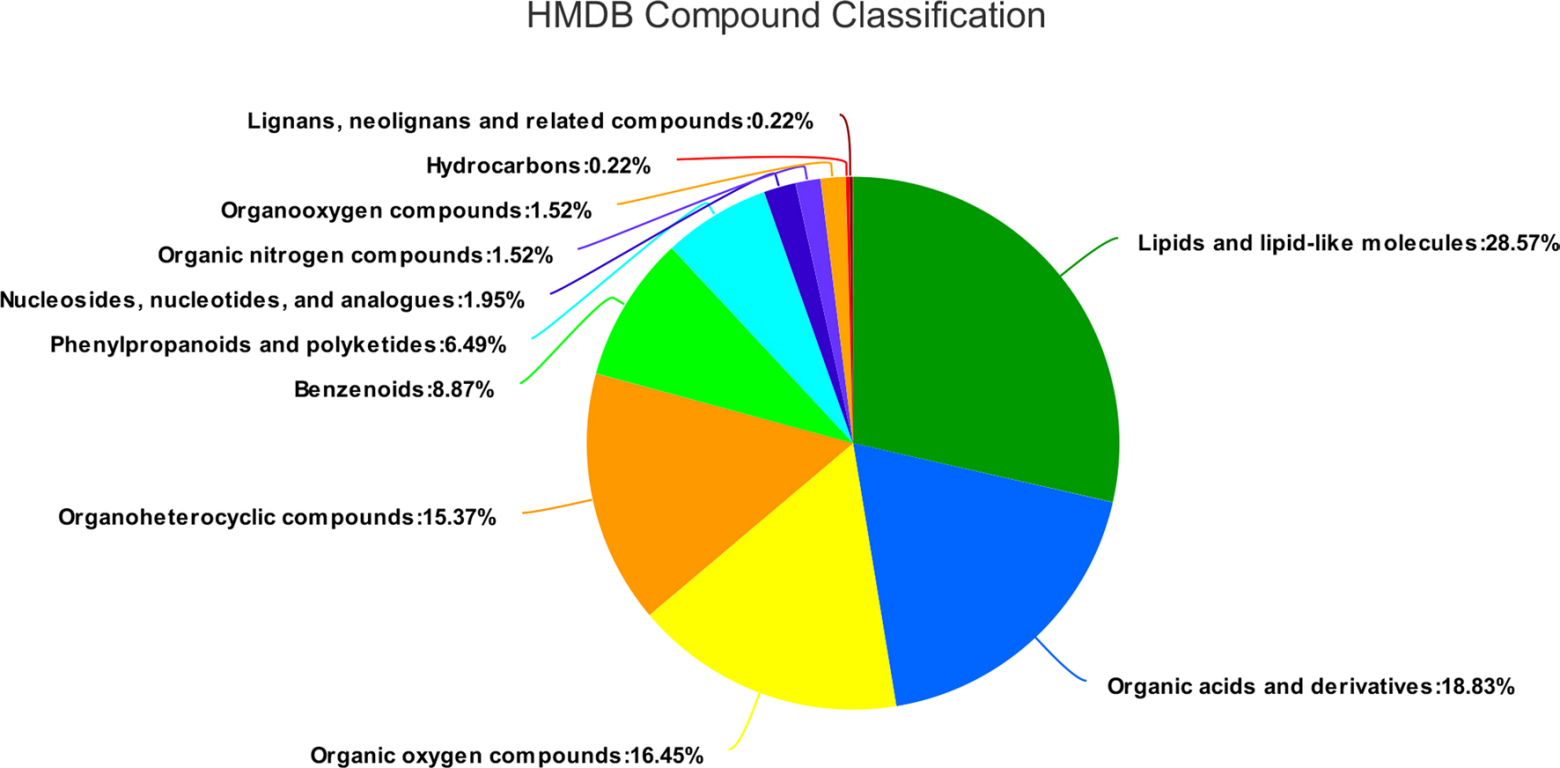
Pie chart showing the percentages of diverse urine metabolites based on counts of HMDB chemical taxonomy (“Super class”). The selected HMDB level (“Super class”) and the percentage of metabolites are displayed in a descending order. Different colors on the pie chart represent different HMDB categories while the area represents relative proportions of metabolites in that category

The taxonomic information of metabolites obtained from the HMDB 4.0 database showed that there were diverse metabolites in urine (Fig. 2). With regards to metabolic concentrations (cationic mode and anionic mode), proportions were: Lipids and lipid-like molecules accounted for 28.57% (132 metabolites), organic acids and derivatives accounted for 18.83% (87 metabolites), organic oxygen compounds accounted for 16.45% (76 metabolites), organoheterocyclic compounds accounted for 15.37% (71 metabolites), benzenoids accounted for 8.87% (41 metabolites), phenylpropanoids and polyketides accounted for 6.49% (30 metabolites), nucleosides, nucleotides, and their analogues accounted for 1.95% (9 metabolites), organic nitrogen compounds accounted for 1.52% (7 metabolites), organooxygen compounds accounted for 1.52% (7 metabolites), hydrocarbons accounted for 0.22% (1 metabolite), while lignans, neolignans and related compounds accounted for 0.22% (1 metabolite).

### Multivariate analysis

**Fig. 3:**
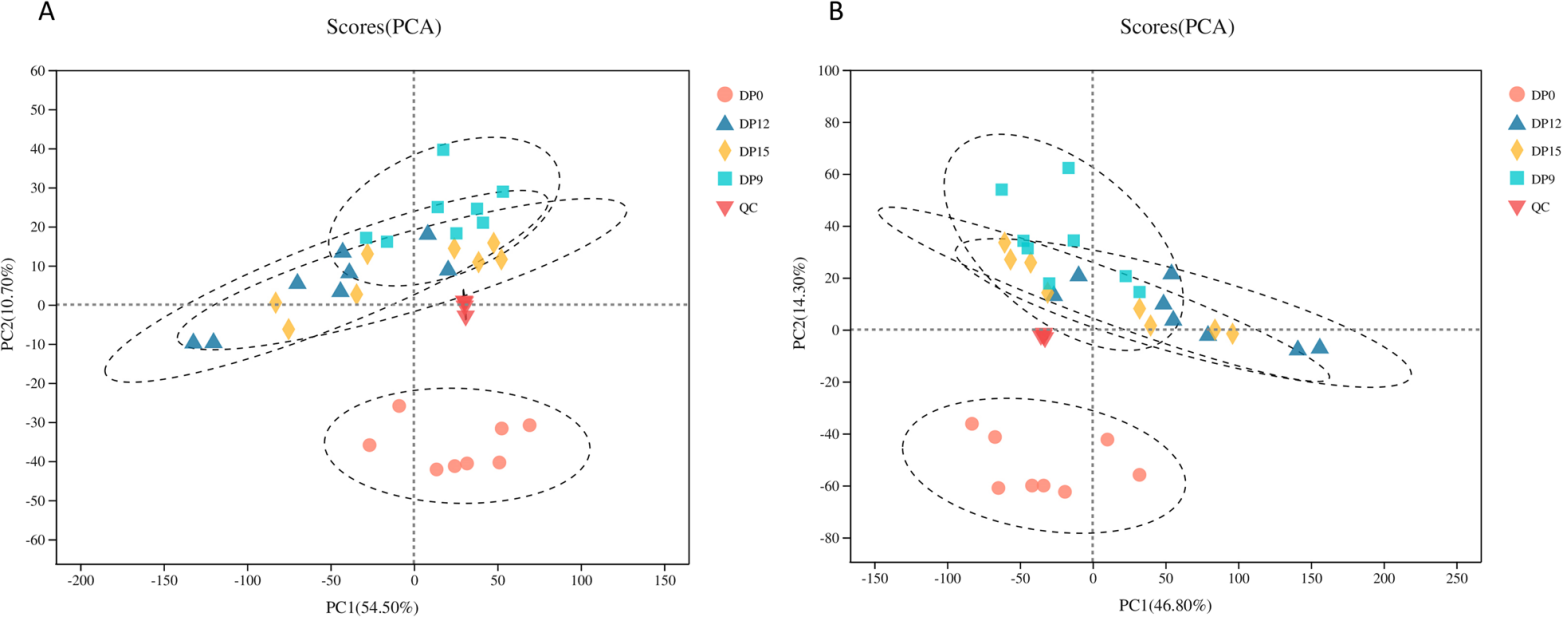
Principle component analysis score plots of metabolites identified in urine during estrus and pregnancy periods. **A** represents positive ions, **B** represents negative ions. Each point on the PCA score chart represents a sample

Principle component analysis (PCA) was performed to observe the consistency of experimental samples and quality control samples. The PCA score curve representing QC samples was tightly clustered, compared to other samples (Fig. 3A, B). The QC results showed that our experiment was stable and the obtained data met the conditions for subsequent statistical analyses.

PCA was used to assess sample separation and aggregation between pregnant and non-pregnant sows. The aggregation points indicated a high similarity of the observed variables, whereas discrete points showed significant differences in observed variables (VIP ≥ 1; ratio ≥ 2 or Ratio ≤ 1/2; q ≤ 0.05). In the positive ion mode, the PCA score showed 54.50% variation at PC1 and 10.70% variation at PC2 (Fig. 3A). In the negative ion mode, the PCA score showed 46.80% variation at PC1 and 14.30% variation at PC2 (Fig. 3B). The results showed that urine in estrus and early pregnancy periods exhibited different metabolic characteristics.

The PCA diagram (Fig. 3) showed that metabolites at 9 days of pregnancy (DP9), 12 days of pregnancy (DP12) and 15 days of pregnancy (DP15) were clustered together, while metabolites at estrus period (DP0) were clustered together. There were little differences in urine metabolism of sows in early pregnancy, however, there were significant differences between pregnancy and estrus. Then, we focused on changes in metabolites during pregnancy and estrus periods.

PCA analysis further showed different metabolic characteristics between the urine of pregnant sows and that of estrous sows, indicating that a significant separation of urine metabolites in sows during estrus and early pregnancy. PCA characterized the separation between observation groups in the experimental models and could not identify specific changes between groups. Therefore, to identify specific differences between groups, PLS-DA, a supervised statistical method of discriminant analysis, was employed. The higher the value of PLS-DA model parameters (R2 and Q2), the higher the reliability of the PLS-DA model. Comparative analysis of 9 days of pregnancy and estrus: The R2 of PLS-DA model in the positive ion mode was 0.819 and Q2 was 0.961 (Additional file 1: Figure S1), while in the negative ion mode, R2 of PLS-DA model was 0.809 and Q2 was 0.978 (Additional file 1: Figure S1). Comparative analysis of 12 days of pregnancy and estrus: The R2 of PLS-DA model in the positive ion mode was 0.821 and Q2 was 0.953 (Additional file 1: Figure S2), while in the negative ion mode, the R2 of PLS-DA model was 0.836 and Q2 was 0.964 (Additional file 1: Figure S2). Comparative analysis of 15 days of pregnancy and estrus: The R2 of PLS-DA model in the positive ion mode was 0.651 and Q2 was 0.968 (Additional file 1: Figure S3), whereas in the negative ion mode, the R2 of PLS-DA model was 0.765 and Q2 was 0.983 (Additional file 1: Figure S3). Generally, findings from the PLS-DA model revealed that, both R2 and Q2 were high in early pregnancy. Based on the parameters of the PLS-DA model, model differences were credible, and the PLS-DA model was used for subsequent analyses.

Furthermore, OPLS-DA was used to analyze metabolites in urine during estrus and pregnancy. Comparative analysis of 9 days of pregnancy and estrus in the positive ion mode revealed OPLS-DA parameters as: R_2X = 0.667, R_2Y = 0.996 and Q_2 = 0.949 (Additional file 1: Figure S4) whereas the negative ion mode had OPLS-DA parameters as: R_2X = 0.486, R_2Y = 0.985 and Q_2 = 0.951 (Additional file 1: Figure S4). Comparative analysis of the 12th day of pregnancy and estrus revealed that in the positive ion mode, OPLS-DA parameters were: R_2X = 0.810, R_2Y = 0.992, Q_2 = 0.946 (Additional file 1: Figure S5), while in the negative ion mode, OPLS-DA parameters were: R_2X = 0.765, R_2Y = 0.995, Q_2 = 0.961 (Additional file 1: Figure S5). Comparative analysis of 15 days of pregnancy and the estrus revealed that in the positive ion mode, OPLS-DA parameters were: R_2X = 0.710, R_2Y = 0.984, Q_2 = 0.948 (Additional file 1: Figure S6). On the other hand, negative ion mode showed OPLS-DA parameters as: R_2X = 0.740, R_2Y = 0.997, Q_2 = 0.965 (Additional file 1: Figure S6). In each comparison, 200 permutation tests were used to validate the OPLS-DA model. The intercept of the OPLS-DA model did not reach the over-fitting threshold (R2Y > 0.4, Q2Y > 0.05). The OPLS-DA score chart showed a clear separation of urine between estrus and pregnancy samples. All Q2 values were greater than 0.4, indicating that the OPLS-DA model used was reliable and achieved consistent modeling as well as prediction results.

### Expression characteristics of differential metabolites during the peri-implantation period

Different metabolites were screened by multivariate statistical analyses of VIP scores obtained via the OPLS-DA model. Compared to the estrus period, a total of 269 differential metabolites were obtained in early pregnancy. Differential metabolites were defined as: VIP ≥ 1; Ratio ≥ 2 or Ratio ≤ 1/2; q ≤ 0.05. Compared to the estrus stage, there were 186 differential metabolites (Additional file 5: Table S4) at 9 days of gestation, 177 differential metabolites (Additional file 6: Table S5) at 12 days of gestation and 169 differential metabolites (Additional file 7: Table S6) at 15 days of gestation. Significantly different metabolites were used to construct heat maps for unsupervised clustering. Heat maps were used to define the different levels of metabolites in urine during estrus and pregnancy stages. Consistent with OPLS-DA results, significant clustering (Additional file 1: Figure S7) was shown in both early pregnancy and during estrus stages.

**Fig. 4:**
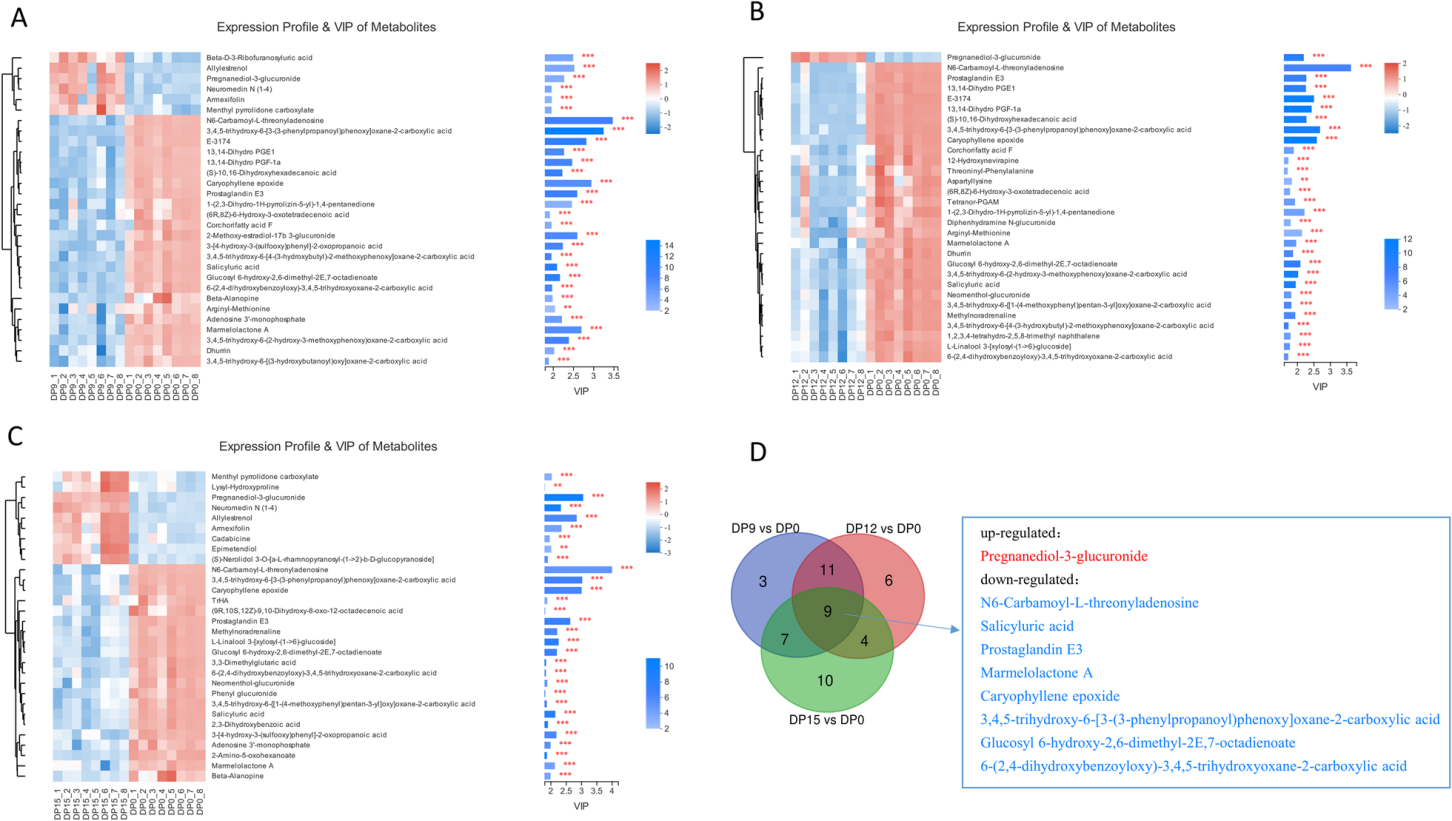
VIP score analysis based on weighted coefficients of the OPLS-DA model used to rank the contribution of top 30 metabolites to the discrimination between estrus and early pregnancy groups. **A** Heat map showing VIP of metabolites between DP9 and DP0, **B** A heat map showing VIP of metabolites between DP12 and DP0, **C** A heat map showing VIP of metabolites between DP15 and DP0, **D** The overlap of metabolites between early pregnancy (DP9, DP12, DP15) and estrus (DP0)

The VIP score, based on the OPLS-DA model represented metabolites that were significantly differentially expressed in the two groups, and those variables with VIP scores greater than 1 were considered important in the classification model. Compared to the estrus stage, the top 30 differentially expressed metabolites in the three phases of early pregnancy were selected for analyses (Fig. 4A–C). Nine metabolites overlapped during early pregnancy and estrus periods (Fig. 4D). Of the nine, one metabolite, Pregnanediol-3-glucuronide (PdG), was up-regulated in pregnancy compared to estrus period. Eight metabolites, including N6-carbamoyl-L-threonyladenosine, salicyluric acid, prostaglandin E3, marmelolactone A, caryophyllene epoxide, 3,4,5-trihydroxy-6-[3-(3-phenylpropanoyl)phenoxy]oxane-2-carboxylic acid, glucosyl 6-hydroxy-2,6-dimethyl-2E,7-octadienoate, and 6-(2,4-dihydroxybenzoyloxy)-3,4,5-trihydroxyoxane-2-carboxylic acid were down-regulated. The significant changes among these metabolites during early pregnancy may be associated with regulation of blastocyst implantation.

### Metabolic pathways for the metabolites

**Fig. 5:**
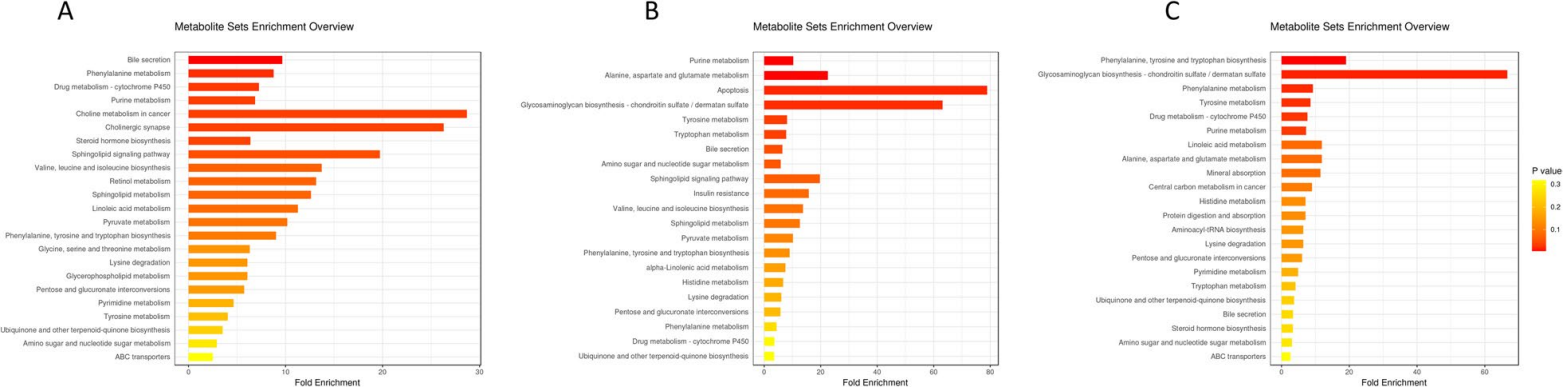
Histogram showing differential metabolites annotated by comparisons to the Kyoto Encyclopedia of Genes and Genomes (KEGG) database. Differential metabolites were classified by KEGG pathway enrichment and significance analysis. Fold enrichment is presented as the ratio of the number of metabolites assigned to the modified pathway by enrichment analysis to the theoretical number of metabolites assigned to the modified pathway by random distribution. The degree of enrichment is indicated by different colors in the histogram, according to the *p* value. Histogram** (A) **shows DP9 versus DP0, histogram** (B)** shows DP12 versus DP0, whereas histogram** (C)** shows DP15 versus DP0

Variable metabolites from DP9 and DP0, DP12 and DP0, DP15 and DP0, were subjected to further biochemical pathway analyses. Functional enrichment showed a significant shift (*p* < 0.05) in expression of molecules associated with amino acid metabolism, lipid metabolism and organic acid production during early pregnancy (Fig. 5). Compared to the estrus stage, at 9 days of pregnancy, the major urinary metabolite enrichment pathways of pregnancy were bile secretion, phenylalanine metabolism, drug metabolism—cytochrome P450, purine metabolism, choline metabolism in cancer, cholinergic synapse, steroid hormone biosynthesis, and sphingolipid signaling pathway (Fig. 5A). Compared to estrus period, at 12 days of pregnancy, the major urinary metabolite enrichment pathways were purine metabolism, alanine, aspartate and glutamate metabolism, apoptosis, glycosaminoglycan biosynthesis—chondroitin sulfate/dermatan sulfate, tyrosine metabolism, tryptophan metabolism, bile secretion, amino sugar as well as nucleotide sugar metabolism, and sphingolipid signaling pathway (Fig. 5B). Relative to the estrus period, at 15 days of pregnancy, the main urinary metabolite enrichment pathways were phenylalanine, tyrosine and tryptophan biosynthesis, glycosaminoglycan biosynthesis—chondroitin sulfate/dermatan sulfate, phenylalanine metabolism, tyrosine metabolism, drug metabolism—cytochrome P450, and purine metabolism (Fig. 5C). In the urinary metabolic pathway, in early pregnancy, a shift in amino acid metabolism was observed in biosynthesis and metabolism of phenylalanine, tyrosine and tryptophan. However, the amino acid metabolic pathway also underwent slight changes at different stages, with phenylalanine predominating on day 9 of pregnancy and tyrosine on days 12 and 15 of pregnancy. In terms of lipid metabolism, in early pregnancy, 17-beta-Estradiol-3-glucuronide, 2-Methoxy-estradiol-17b 3-glucuronide, 4-oxo-Retinoic acid, 9,10,13-TriHOME, cholestane-3,7,12,25-tetrol-3-glucuronide, pregnanediol-3-glucuronide, SM(d18:0/16:1(9Z)) and traumatic acid were enriched.

**Fig. 6:**
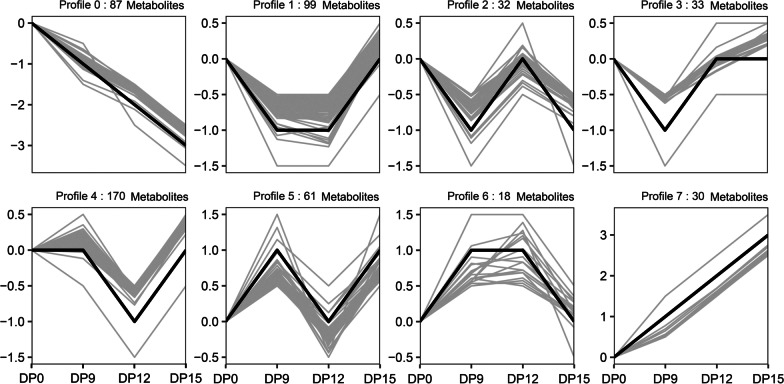
Relative expression levels of urinary metabolites at 4 different stages of porcine gestation. All metabolites were clustered into 8 soft partitioned clusters based on expression patterns

Furthermore, to characterize the expression profiles of 530 metabolites, expression data (DP0 to DP9, DP9 to DP12, and DP12 to DP15) were normalized to 0, log_2_^(DP9/DP0)^, log_2_^(DP12/DP0)^, and log_2_^(DP15/ DP0)^, as analyzed by the Short Time-series Expression Miner (STEM), the 530 metabolites were clustered into eight profiles (0–7) (Fig. 6, Additional file 8: Table S7). The metabolite, PdG, was clustered in profile 7, and its expression levels continued to increase during the peri-implantation period. The eight metabolites that were significantly down-regulated during the peri-implantation stage were clustered in profile 0 (5 metabolites: salicyluric acid, prostaglandin E3, marmelolactone A, caryophyllene epoxide, and glucosyl 6-hydroxy-2,6-dimethyl-2E,7-octadienoate), profile 1 (2 metabolites: 3,4,5-trihydroxy-6-[3-(3-phenylpropanoyl)phenoxy]oxane-2-carboxylic acid, 6-(2,4-dihydroxybenzoyloxy)-3,4,5-trihydroxyoxane-2-carboxylic acid), and profile 4 (1 metabolite: N6-carbamoyl-L-threonyladenosine) (Additional file 8: Table S7). These metabolic changes in early pregnancy reflect changes in nutritional needs of the embryo and uterus, as well as the metabolic state of the mother.

## Discussion

We used the MS-based approach and reliable metabolite identification to detect urine metabolites through UPLC Q-TOF/MS analysis. Urine was obtained from eight sows in estrus and early pregnancy periods and examined to determine changes in metabolite levels of different pathways associated with early pregnancy. A total of 269 differential metabolites were identified in the early pregnancy period while compared to estrus period, a total of 186 different metabolites were found at 9 days of gestation, 177 different metabolites at 12 days of gestation, and 169 different metabolites at 15 days of gestation. These differential metabolites represent changes in various metabolic processes, such as amino acid and lipid metabolism as well as organic acid contents. One of the metabolites for choline is vitamin B. Choline is an essential nutrient for mammals, an important component of phospholipids in cell membranes and one of the raw materials for the body's synthesis of glycosaminoglycans [28]. The metabolite is involved in lipid metabolism, brain development and fetal development [29, 30]. The choline derivative, phosphatidylcholine, is a major constituent of all cell membranes and is required for the biosynthesis of lipoproteins, including very low-density lipoproteins (VLDLs), which facilitate hepatic exports of lipids [31]. Choline regulates growth and metabolism in animals [32, 33]. During embryogenesis, choline is essential for the growth of cellular structures [29]. Choline kinase is detected at all stages of embryonic development, and its activity increases during development and qualitatively parallels the extent of phosphorylcholine formation in intact embryos [34]. Studies have shown that choline chloride alters development, specific gene expression and DNA methylation in bovine embryos cultured in vitro in a concentration-dependent manner. The role of choline chloride in promoting DNA methylation in the blastocyst is not only as a methyl donor, but may also be due to the role of choline itself or choline metabolites in a variety of functions including cell signalling [35]. Transcriptional coding for enzymes involved in the synthesis of these choline derivatives is present during the peri-implantation phase in mammals [36, 37], and there is evidence that these pathways are important for preimplantation development. In mice, addition of acetylcholine to the culture medium allows embryos to develop from the 2-cell block zone to the blastocyst stage [38], and knockdown of the choline kinase gene (the first enzyme in the phosphatidylcholine synthesis pathway) results in the death of mouse embryos between 3.5 and 7.5 days of development [39]. In addition, studies have confirmed that before the implantation, compared with the blank group, the implantation rate of the choline-added rat group was higher than that of the blank group [40]. During pregnancy, choline ingested by the mother is transported through the placenta to the fetus [41]. Adequate choline intake during pregnancy is essential for proper fetal development [42]. Supply of maternal choline to the fetus plays an important role in fetal brain development, membrane biosynthesis, and neurotransmission [31]. In a research involving rat models, prenatal choline supplementation preserved a sustained memory state of rats and protected offsprings from developing memory defects [43]. In pigs, dietary choline deficiency can cause significant changes in plasma choline metabolites at the end of lactation. These changes in concentrations of nutrients such as choline may affect the development of early newborns [44]. Although examination was not performed on natural pregnant sows, we postulate that this is one of the explanations for the increase in urine choline levels in sows during pregnancy. Pregnancy and lactation are the periods when maternal reserves of choline are depleted. At the same time, the availability of choline for normal development of the brain is critical because it influences lifelong memory enhancement [45]. The broad role and diverse involvement of choline in cells suggests the importance of its pathway to regulate embryonic development and prepare for implantation.

Of the various differentially expressed metabolites, pregnanediol-3-glucuronide (PdG) was highly expressed in all the three phases of early pregnancy. The PdG metabolite plays an important role in regulation of mammalian pregnancy. Volkery et al. investigated the levels of PdG in plasma, saliva, milk, and urine of alpaca without pregnancy and throughout pregnancy. The concentrations of PdG in plasma, milk, and urine were significantly higher than those in non-pregnant alpaca [46]. The ovaries of mammals are luteinized once they are pregnant, and the corpus luteum dissolves at the end of pregnancy [47]. Ecochard et al. characterized the variabilities in hormonal profiles during the luteal phase in normal cycles. They found that PdG levels increased with early lutealization, high levels of PdG were maintained in the mid-luteal phase which decreased gradually to a low level in the late luteal phase (luteolysis) during the non-pregnant state [48]. The occurrence of normal and low luteal PdG levels are potential markers of luteal phase abnormality [49]. In a study by Rene Leiva et al., assessment of urine PdG levels was used as an auxiliary means to confirm the effectiveness of ovulation [50]. Its concentration was found to be positively correlated with the number of pregnant embryos in feces of female golden monkeys [51], while its accuracy and sensitivity as a pregnancy test marker were validated in monitoring pregnancies in *Mus musculus* [52]. In this study, we found that PdG was highly expressed in the urine of sows during early pregnancy (Fig. 6, Profile 7), in tandem with levels in various body fluids during early pregnancy in other mammals. PdG, the main terminal metabolite of progesterone, plays an important role in physiological processes like female menstrual cycle, pregnancy embryogenesis and maternal immune responses in some mammals [53]. In this study, sow urinary PdG levels showed a continuous increasing trend during the peri-implantation period, which may be associated with embryonic implantation and development, as the need for pregnancy maintenance increased.

Pathways associated with metabolites identified at each pregnancy phase were analysed. The results showed that tyrosine metabolism, phenylalanine metabolism, and tryptophan metabolism play an important role in pregnancy. Liu et al. performed a longitudinal analysis of urine from 50 healthy pregnant women and found own-regulation of tyrosine metabolism during pregnancy [16]. During pregnancy, tyrosine metabolism can be formed by phenylalanine hydroxylation [54] and these two pathways are also reflected in this study (Fig. 5). Maternal tyrosine intake during gestation plays a pre-regulatory role in the development of dopaminergic systems in offsprings after birth [55]. McBride et al. determined the reference ranges for phenylalanine and tyrosine levels in healthy pregnancy. They reported that phenylalanine and tyrosine levels rapidly declined in early pregnancy and remained relatively stable in mid- and late pregnancy [56]. Tryptophan is an indispensable amino acid in mammalian protein synthesis. Tryptophan metabolism within different tissues is associated with numerous physiological functions. The liver regulates tryptophan homeostasis by degrading excess tryptophan. Tryptophan degradation into kynurenine by immune cells plays a crucial role in regulation of immune responses during infections, inflammation, and pregnancy [57]. Tryptophan enhances maternal protein synthesis, fetal growth and development and improves kynurenines inhibition of fetal rejection during gestation in mammals [58]. Tryptophan metabolism during mammalian gestation can be achieved through multiple pathways [59]. For instance, through the kynurenine pathway, where tryptophan is converted to kynurenine by the action of indoleamine 2-3 deoxygenase and tryptophan-2,3-double-oxygenase. Then, kynurenine is converted to kynurenic acid by the actions of kynurenine aminotransferase (KAT). In cases of depression, kynurenic acid may have neuro-protective effects [60]. The tryptophan metabolic pathway also generates 3-hydroxyanthranilic acid (HA), an effective antioxidant, intra-cellularly via kynurenic acid, in the extrahepatic tissue [61]. Tryptophan and its metabolites are effective in scavenging free radicals (including reactive oxygen and reactive chlorine), therefore, they can be targeted in the placenta as potent antioxidants [62]. In this study, compared to the estrus period, various differentially expressed metabolites in early pregnancy were found to be enriched in the amino acid metabolism pathway. This change may be required for embryonic development or immune regulation as in other animals. We did not exhaust differential metabolites and related pathways (including some down-regulated metabolites), therefore, additional studies should be conducted in this area. Furthermore, there are some limitations of this study, one being whether the relevant metabolite levels correlate with the number of successful embryos implanted to screen sows with different fecundity levels, and the other being whether the urinary metabolites of sows without implantation after insemination also have relevant changes is an area that deserves further research. In addition, in commercial farms, the collection of urine samples immediately put into liquid nitrogen preservation may not working in daily practise. It can be taken to add 0.42% sodium azide preservative at the final concentration within 2 h of urine sample collection, and store it at − 80 °C[63].

## Conclusions

Urinary metabolomics of sows during estrus and early pregnancy were determined by UHPLC. A total of 269 urinary metabolites, including lipids and organic acids were identified in early pregnancy. In particular, PdG levels were consistently highly expressed at each pregnancy phase of early pregnancy. As the main terminal metabolite of progesterone, its high expressions are necessary to maintain pregnancy. To the best of our knowledge, this is the first investigation of urinary metabolic changes in sows during early pregnancy. Our findings elucidate on maternal urinary changes caused by intrauterine embryonic implantation during early pregnancy in sows. Moreover, they form a basis for evaluating the molecular mechanisms involved in maternal–fetal communication during embryonic implantation and nutritional requirements of early pregnancy pigs.

## Supplementary Information

**Additional file 1.Table S1**. All the positive ion features.

**Additional file 2.** **Table S2**. All the negative ion features.

**Additional file 3.** **Table S3**. Metabolites identified at high confidence level.

**Additional file 4.** **Table S4**. MetabsetVip_DP9_vs_DP0.

**Additional file 5.** **Table S5**. MetabsetVip_DP12_vs_DP0.

**Additional file 6.** **Table S6**. MetabsetVip_DP15_vs_DP0.

**Additional file 7.** **Table S7**. Expression profiles(0-7).

**Additional file 8.** Supplementary Figures (Figure S1 to S7).

## Data Availability

The complete dataset for this study can be accessed at: https://www.ebi.ac.uk/metabolights/MTBLS2135 [64].

## Abbreviations

UPLC–MS/MS: Ultra-performance liquid chromatography–tandem mass spectrometry
RT: Retention time
m/z: Mass-to-charge ratio
DDA: Data dependent acquisition
PdG: Pregnanediol-3-glucuronide
DP0: Day 0 of pregnancy
DP9: Day 9 of pregnancy
DP12: Day 12 of pregnancy
DP15: Day 15 of pregnancy
PCA: Principle component analysis
PLS-DA: Partial least squares-discriminant analysis
OPLS-DA: Orthogonal partial least square-discriminant analysis
VIP: Variable importance in projection
QC: Quality control sample
STEM: Short time-series expression miner
