# Supplementary material for: Urinary metabolomics reveals the biological characteristics of early pregnancy in pigs
Source: Porcine Health Manag. 2022 Mar 21;8:14. doi: 10.1186/s40813-022-00256-z (PMC8935750; doi:10.1186/s40813-022-00256-z)
Supplement: Supplementary file 1 — Additional file 1.Table S1. All the positive ion features. [file 40813_2022_256_MOESM1_ESM.pdf]

Supplementary Figures

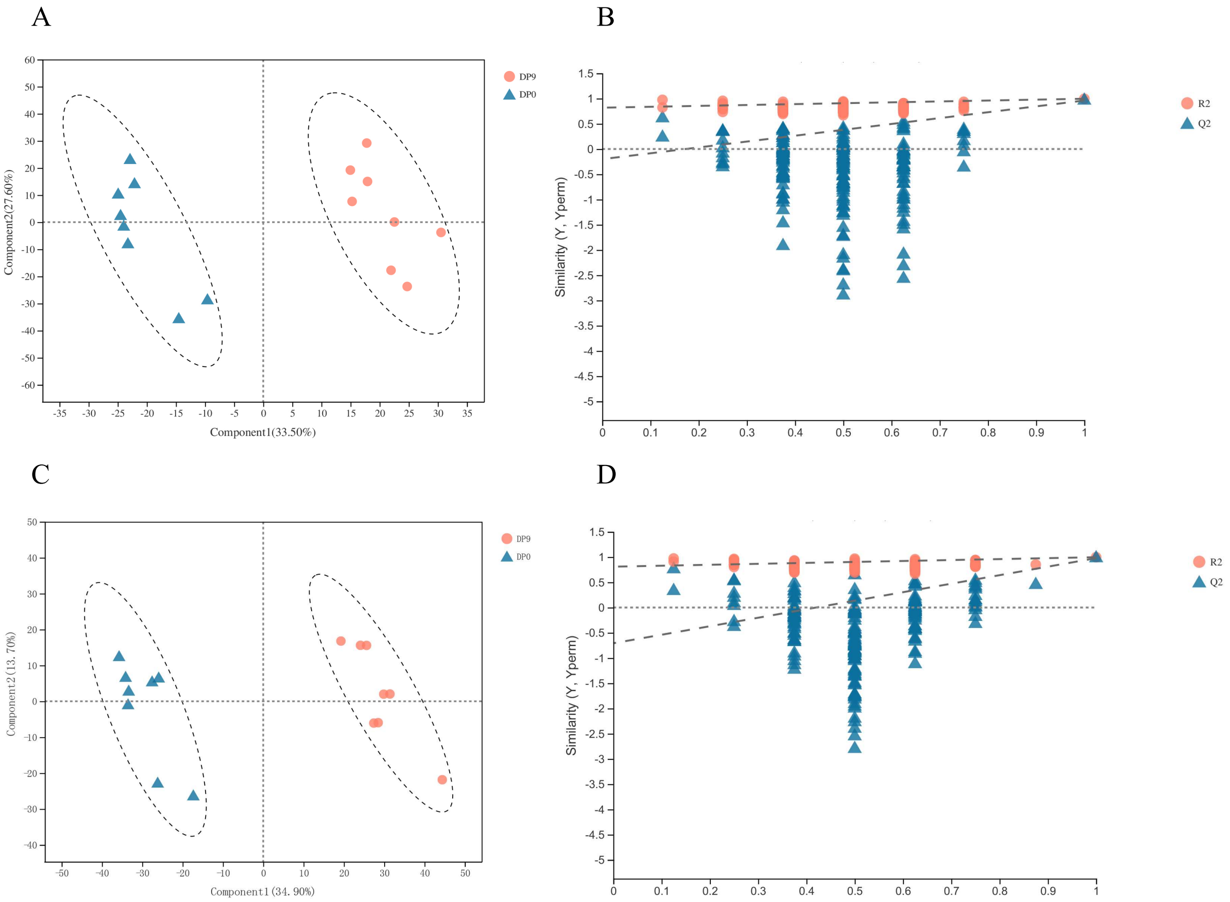

Figure S1. Partial least squares-discriminant analysis (PLS-DA) score plots of metabolites identified in urine during estrus and 9 days of pregnancy. (A) Positive ions, (B) PLS-DA model validation under positive ions, (C) Negative ions, (D) PLS-DA model validation under negative ions. The left points represent Q2 and R2 of 200 permutation tests, and the right two points represent Q2 and R2 of the real PLS-DA model. Both values are higher than those in the permutation tests, respectively, indicating the model’s robustness.

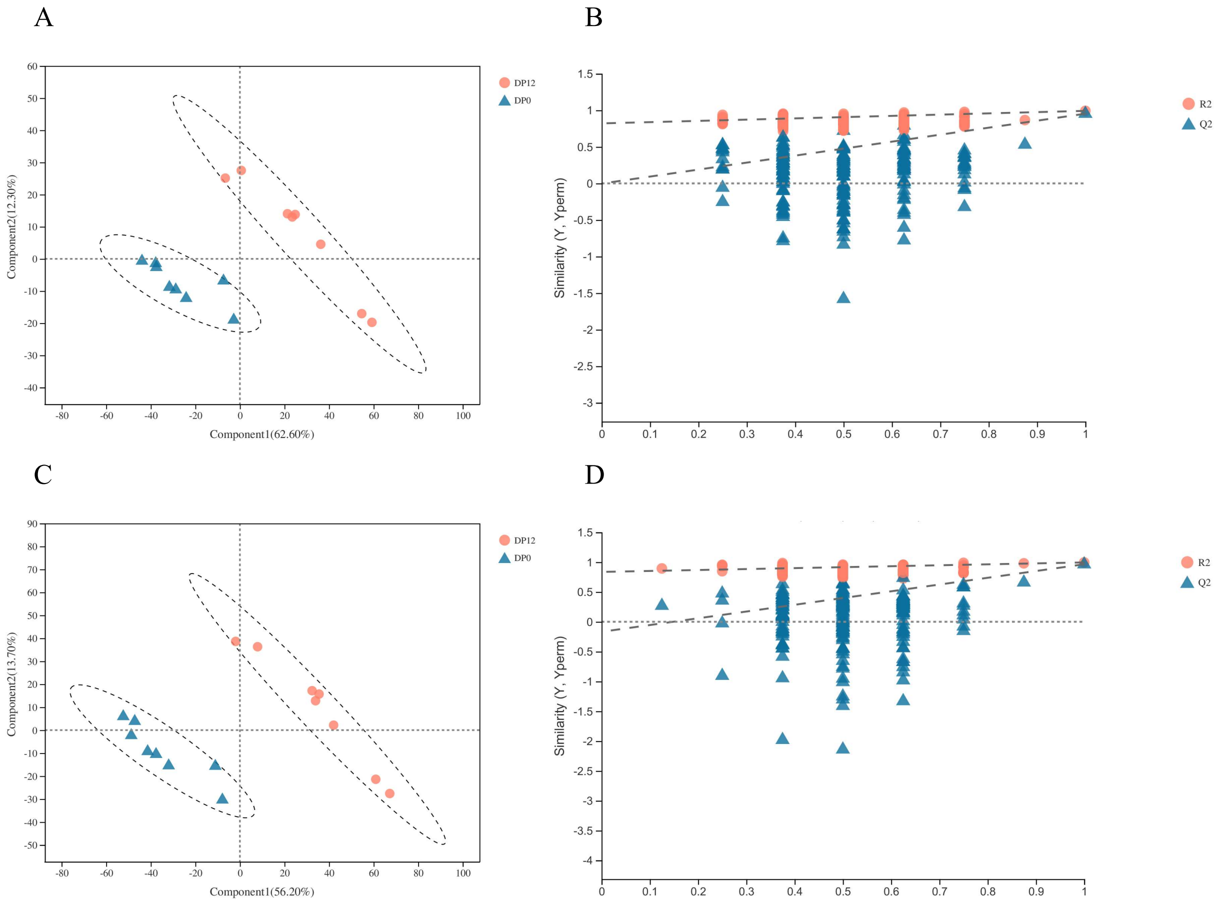

Figure S2. Partial least squares-discriminant analysis (PLS-DA) score plots of metabolites identified in urine during estrus and 12 days of pregnancy. (A) Positive ions, (B) PLS-DA model validation under positive ions, (C) Negative ions, (D) PLS-DA model validation under negative ions. The left points represent Q2 and R2 of 200 permutation tests, and the right two points represent Q2 and R2 of the real PLS-DA model. Both values are higher than those in the permutation tests, respectively, indicating the model’s robustness.

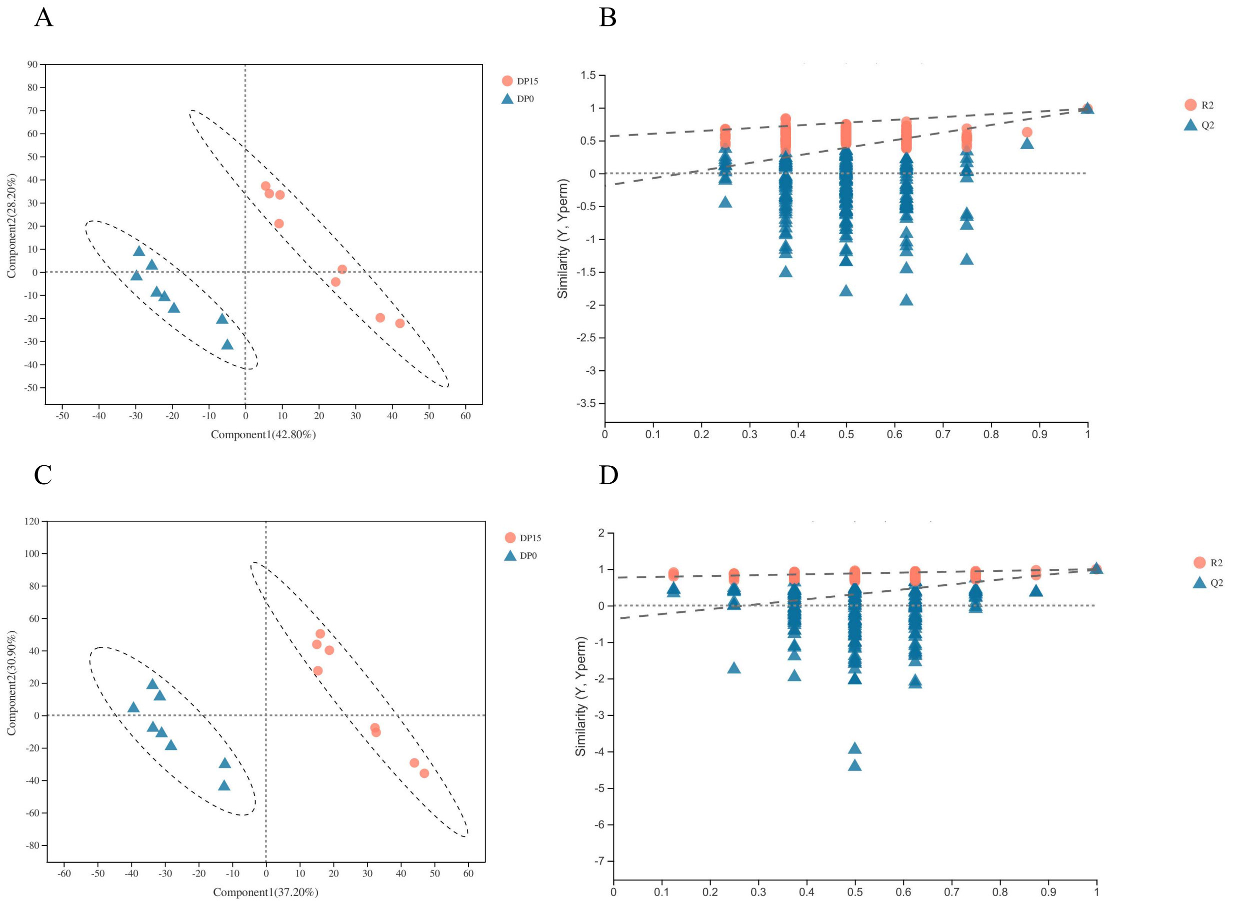

Figure S3. Partial least squares-discriminant analysis (PLS-DA) score plots of metabolites identified in urine during estrus and 15 days of pregnancy. (A) Positive ions, (B) PLS-DA model validation under positive ions, (C) Negative ions, (D) PLS-DA model validation under negative ions. The left points represent Q2 and R2 of 200 permutation tests, and the right two points represent Q2 and R2 of the real PLS-DA model. Both values are higher than those in the permutation tests, respectively, indicating the model's robustness.

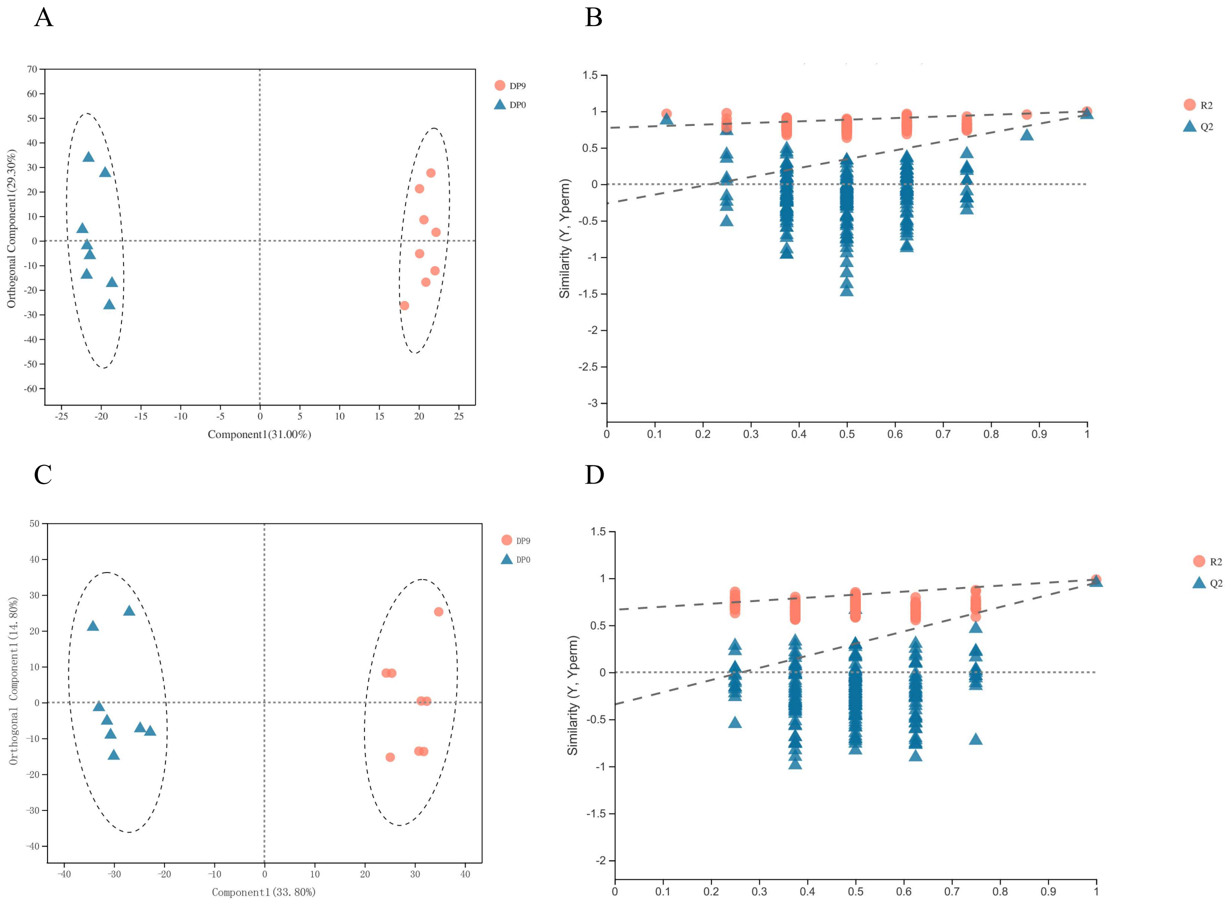

Figure S4. Orthogonal partial least squares-discriminant analysis (OPLS-DA) score plots of metabolites detected in urine during estrus and 9 days of pregnancy. (A) Positive ion, (B) OPLS-DA model validation under positive ions, (C) Negative ion, (D) OPLS-DA model validation under negative ions.

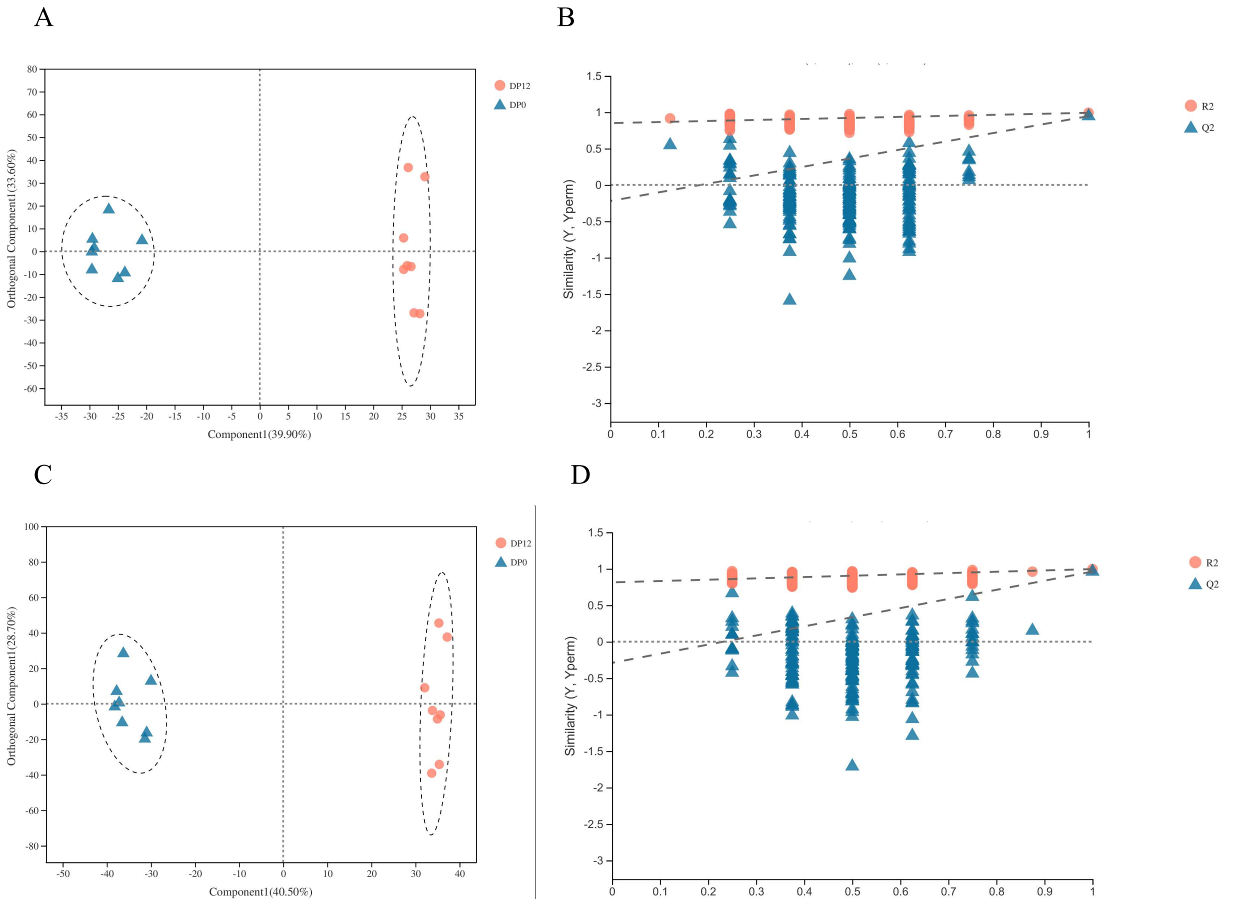

Figure S5. Orthogonal partial least squares-discriminant analysis (OPLS-DA) score plots of metabolites identified in urine during estrus and 12 days of pregnancy. (A) Positive ion, (B) OPLS-DA model validation under positive ions, (C) Negative ion, (D) OPLS-DA model validation under negative ions.

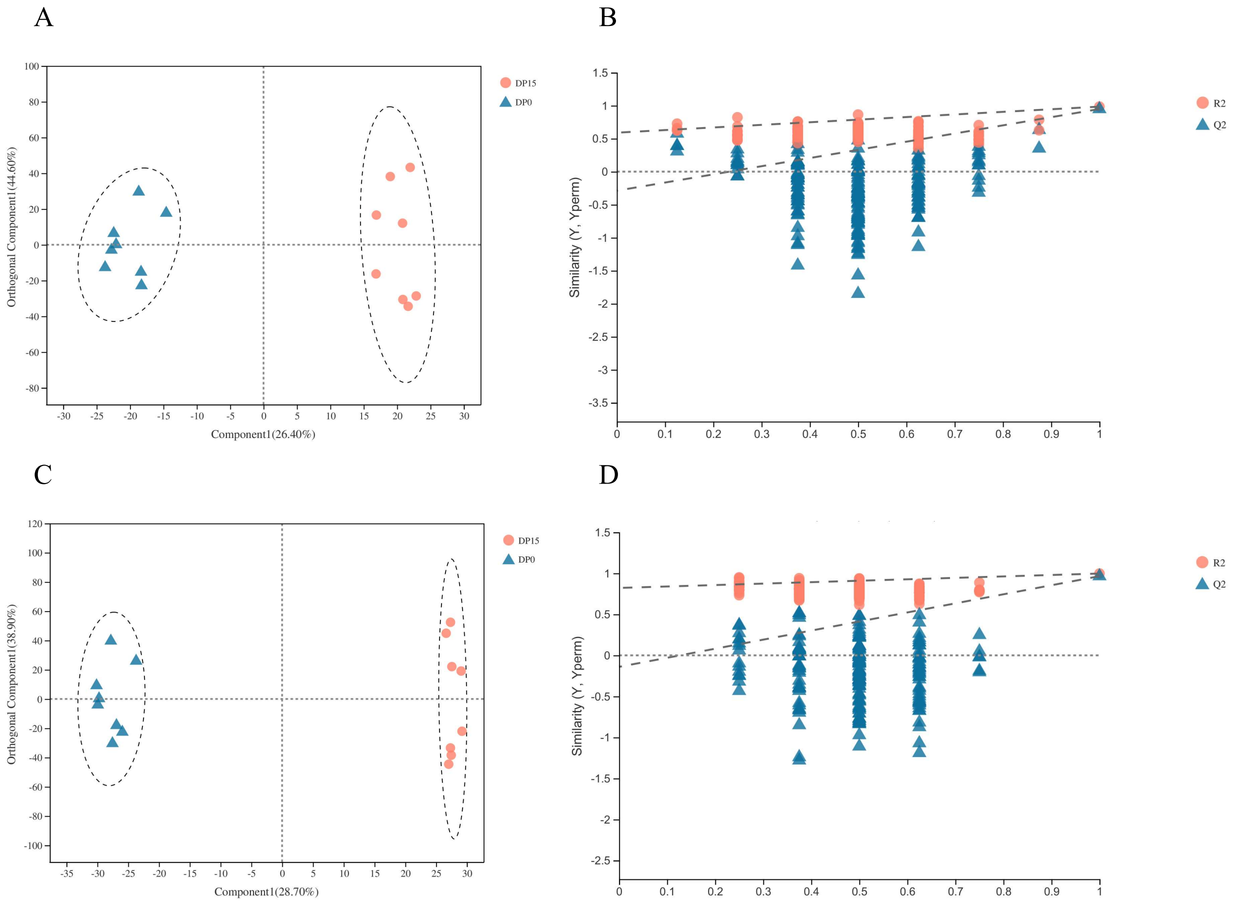

Figure S6. Orthogonal partial least squares-discriminant analysis (OPLS-DA) score plots of metabolites identified in urine during estrus and 15 days of pregnancy. (A) Positive ion, (B) OPLS-DA model validation under positive ions, (C) Negative ion, (D) OPLS-DA model validation under negative ions.

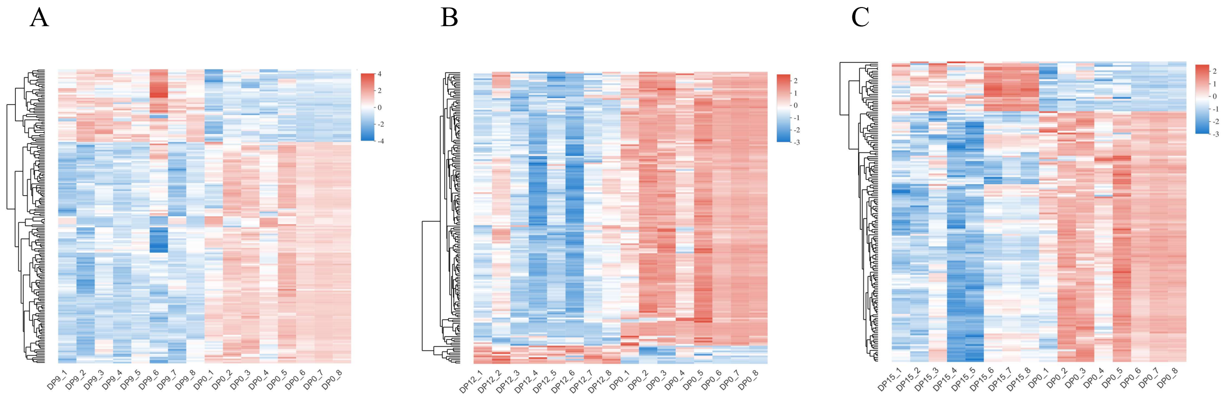

Figure S7. A heatmap of the metabolites identified in the urine of pregnant and non-pregnant sows. (A) DP9 compared to DP0, (B) DP12 compared to DP0, (C) DP15 compared to DP0.
